# Risk Factors for Congenital Syphilis Transmitted from Mother to Infant — Suzhou, China, 2011–2014

**DOI:** 10.15585/mmwr.mm6810a4

**Published:** 2019-03-15

**Authors:** Yajie Wang, Minzhi Wu, Xiangdong Gong, Liang Zhao, Jing Zhao, Chuanwu Zhu, Chancong Gong

**Affiliations:** ^1^Institute of Dermatology, Chinese Academy of Medical Sciences and Peking Union Medical College, Nanjing, China; ^2^Dermatology Hospital of Southern Medical University, Guangzhou, China; ^3^The Fifth People’s Hospital of Suzhou, Suzhou, China.

Mother-to-child transmission of syphilis remains a major global public health issue, and elimination of congenital syphilis is one of the millennium development goals of the World Health Organization (*1*). In 2012, an estimated 930,000 maternal syphilis infections caused 350,000 adverse pregnancy outcomes, including 143,000 early fetal deaths and stillbirths, 62,000 neonatal deaths, 44,000 preterm or low-weight births, and 102,000 infected infants worldwide (*2*). In China, the number of congenital syphilis cases reported annually increased from 468 in 2000 to 10,032 in 2013; the corresponding national congenital syphilis incidence rate increased nearly 26-fold, from 2.6 cases per 100,000 live births in 2000 to 69.9 in 2013 (*3*,*4*). To examine risk factors for mother-to-child transmission of syphilis, a cohort of pregnant women with a new syphilis diagnosis and their live-born infants was recruited during July 2011–July 2014 in Suzhou, in eastern China. Multivariable logistic regression results demonstrated that gestational age >36 weeks at the time of maternal syphilis diagnosis, higher maternal titers of rapid plasma reagin (RPR) and higher *Treponema pallidum* particle agglutination assay (TPPA) titers are risk factors for congenital syphilis. Among women with syphilis diagnosed at >36 weeks’ gestational age, three quarters were migrant women. Recommendations for strengthening community and provider education about mother-to-child transmission of syphilis, early diagnosis and timely treatment of syphilis in pregnancy, and improving and providing access to prenatal care and screening migrant pregnant women with temporary residence status might reduce the incidence of congenital syphilis in China.

From July 2011 through July 2014, a cohort of 189 pregnant women with a new diagnosis of syphilis was recruited at the Fifth People’s Hospital of Suzhou. According to the national diagnostic criteria for syphilis (*5*), only women who had both a reactive RPR (nontreponemal) test and a reactive TPPA (treponemal) test were included in the study; the stages of maternal syphilis were defined as primary, secondary, tertiary, or latent. Information about demographic, clinical, and laboratory characteristics of each woman was obtained, as well as history and current status of syphilis of her spouse (tested with TPPA and RPR at the time of diagnosis of syphilis in the pregnant woman). Timing of the mothers’ infection with syphilis was not ascertained. Migrant status was determined using questionnaires during face-to-face interviews and verified by checking identification documents. Women with human immunodeficiency virus (HIV) coinfection, women who declined to participate, who were lost to follow-up, or who experienced a fetal death or stillbirth were excluded.

Clinical, laboratory, and treatment data regarding the women’s newborn infants were recorded, and infants were followed up at ages 3, 6, 9, 12, 15, and 18 months. Infants who were negative for both RPR and TPPA at any time were considered to not have congenital syphilis, and follow-up was discontinued; if infants were not negative for both RPR and TPPA, they were followed up until age 18 months. Because *T. pallidum*–specific immunoglobulin G can be passively transferred from the mother to newborn, a reactive serologic test at birth does not necessarily indicate that the infant is infected. If the infant is not infected, passively transferred immunoglobulin G antibodies typically decline to undetectable levels by age 15 months, whereas in infected infants, treponemal tests can remain positive for life, even with effective therapy. Therefore, a case of congenital syphilis was considered confirmed if an infant had a reactive TPPA test at age 18 months (*6*). All patients with maternal or congenital syphilis were treated immediately, according to the national treatment guidelines for syphilis^†^ (*7*).

Within the maternal cohort, a nested case-control study was conducted to examine the risk for transmission of syphilis from mother to child. Infants with congenital syphilis were classified as case-patients, and those without congenital syphilis were classified as controls. Pearson’s chi-squared test or Fisher’s exact test was used to compare differences in maternal and paternal demographic and laboratory characteristics between cases and controls. Among cases and controls, the relative odds were assessed using univariate and multivariable exact logistic regression analyses, computing unadjusted odds ratios (ORs) and adjusted ORs (aORs) with 95% confidence intervals (CIs). The multivariable exact logistic regression model was built using a forward selection procedure (at p<0.25) (*8*). SAS software (version 9.2; SAS Institute) was used for all statistical analyses. All p-values were two-sided, with values <0.05 being considered statistically significant.

A total of 189 pregnant women with a new diagnosis of syphilis were identified during the study period. Among these women, none was HIV-positive or refused to participate, four (2.1%) experienced a fetal death or stillbirth, and 30 (15.9%) were lost to follow-up, leaving 155 pregnant women (82.0% of the original cohort) and their 155 live-born infants for analysis. By the end of follow-up, 27 infants (17.4%; CI = 12.1%–24.0%) had received a diagnosis of congenital syphilis (Figure).

**FIGURE:**
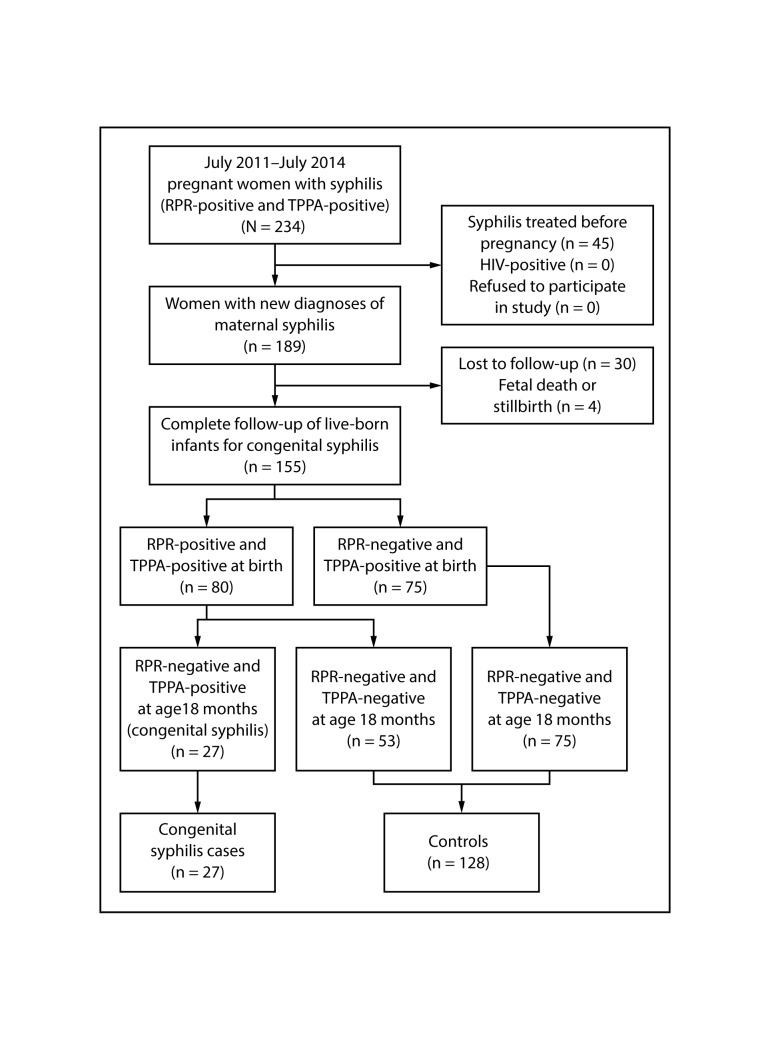
Selection of participants for the cohort, cases, and controls in the congenital syphilis nested case-control study — Suzhou, China, 2011–2014 **Abbreviations:** RPR = rapid plasma reagin; TPPA = *Treponema pallidum* particle agglutination.

Univariate logistic regression analysis indicated that delivery of an infant with congenital syphilis was significantly more likely among migrant women (OR = 4.9; CI = 1.7–17.7) and women who received a diagnosis of maternal syphilis after 36 weeks’ gestational age (OR = 24.1; CI = 3.6–≥1,000.0) (Table 1). Among 60 mothers with syphilis diagnosed at >36 weeks’ gestational age, 45 (75.0%) were migrant women; and among 82 migrant women with syphilis, 54.9% received a diagnosis at >36 gestational weeks. Every twofold increase of maternal RPR titers and TPPA titers increased the risk for mother-to-child transmission of syphilis (OR = 2.6 [RPR]; OR = 1.6 [TPPA]). Compared with biologic fathers who did not have syphilis, infants whose biologic fathers had syphilis had an increased likelihood of having congenital syphilis (OR = 3.7; CI = 1.4–9.9).

**TABLE 1:** Characteristics of mothers with syphilis and infants’ biologic fathers associated with congenital syphilis (univariate analysis) — Suzhou, China, 2011–2014

Characteristic	Women (N = 155)	Infants with congenital syphilis, no. (%)	Unadjusted OR (95% CI)*	P-value
**Age group (yrs)**				
20–24	55	15 (27.3)	1.0	Referent
25–29	45	5 (11.1)	0.3 (0.1–1.1)	0.075
30–34	29	3 (10.3)	0.3 (0.1–1.3)	0.121
≥35	26	4 (15.4)	0.5 (0.1–1.8)	0.373
**Marital status**				
Single	9	2 (22.2)	1.0	Referent
Currently married	140	24 (17.1)	0.7 (0.1–7.6)	0.976
Formerly married	6	1 (16.7)	0.7 (0.0–17.7)	1.000
**Highest level of education completed**				
High school or more	47	4 (8.5)	1.0	Referent
Middle school	83	19 (22.9)	3.2 (1.0–13.7)	0.061
Primary school or less	25	4 (16.0)	2.0 (0.3–12.0)	0.557
**Employed**				
Yes	28	2 (7.1)	1.0	Referent
No	127	25 (19.7)	3.2 (0.7–29.3)	0.178
**Migrant resident**				
No	73	5 (6.8)^†^	1.0	Referent
Yes	82	22 (26.8)	4.9 (1.7–17.7)	0.002
**History of syphilis (father)**				
No	78	9 (11.5)	1.0	Referent
Yes	77	18 (23.4)	2.3 (0.9–6.4)	0.082
**Current syphilis status (father)**				
Negative	104	12 (11.5)^†^	1.0	Referent
Positive	40	13 (32.5)	3.7 (1.4–9.9)	0.009
Unknown	11	2 (18.2)	1.7 (0.2–9.7)	0.797
**Gestational age at syphilis diagnosis (wks)**				
≤12	38	1 (2.6)^§^	1.0	Referent
13–24	36	1 (2.8)	1.1 (0.0–85.2)	1.000
25–36	21	1 (4.8)	1.8 (0.0–149.1)	1.000
>36	60	24 (40.0)	24.1 (3.6–≥1,000.0)	<0.001
**Maternal syphilis stage**				
Primary and secondary	9	2 (22.2)	1.0	Referent
Latent	146	25 (17.1)	0.7 (0.1–7.6)	0.973
**Syphilis treatment during pregnancy** ^¶^				
Benzathine penicillin	91	4 (4.4)	1.0	Referent
Ceftriaxone	7	1 (14.3)	3.6 (0.1–44.7)	0.630
Azithromycin	5	1 (20.0)	5.3 (0.1–75.0)	0.478
**Mode of delivery**				
Vaginal	91	12 (13.2)	1.0	Referent
Cesarean	64	15 (23.4)	2.0 (0.8–5.1)	0.151
**RPR and TPPA titers**				
Log_2_ (titers of RPR)**	—	—	2.6 (1.9–3.8)	<0.001^§^
Log_2_ (titers of TPPA)^††^	—	—	1.6 (1.3–2.0)	<0.001^§^

**Abbreviations:** CI = confidence interval; OR = odds ratio; RPR = rapid plasma reagin; TPPA = *Treponema pallidum* particle agglutination.

* Confidence intervals and p-values from univariate exact logistic regression.

^†^ p<0.05 (Pearson’s chi-squared or Fisher’s exact test for categorical variables and exact test for logistic regression coefficients).

^§^ p<0.001 (Pearson’s chi-squared or Fisher’s exact test for categorical variables and exact test for logistic regression coefficients).

^¶^ Fifty-two women who received a diagnosis of syphilis and were treated at delivery were excluded in analysis of syphilis treatment during pregnancy.

** RPR titers of pregnant women with syphilis = 1–128.

^††^ TPPA titers of pregnant women with syphilis = 80–40,960.

Multivariable logistic regression demonstrated that mothers with syphilis diagnosed at >36 weeks’ gestational age were approximately 25 times more likely to deliver a baby with congenital syphilis than were women who received a diagnosis of syphilis at gestational age ≤12 weeks (aOR = 25.0; CI = 2.5–≥1,000.0) (Table 2). Every twofold increase of maternal RPR titers approximately doubled the odds of delivering an infant with congenital syphilis (aOR = 1.7; CI = 1.2–2.6); similar results were found for TPPA titers (aOR = 1.6; CI = 1.2–2.3).

**TABLE 2:** Risk factors associated with congenital syphilis among 155 women with syphilis (multivariable analysis) — Suzhou, China, 2011–2014

Characteristic	Adjusted OR (95% CI)	P-value
**Gestational age at syphilis diagnosis (wks)**		
≤12	1.0	Referent
13–24	1.6 (0.0–138.1)	1.000
25–36	1.0 (0.0–90.1)	1.000
>36	25.0 (2.5–≥1,000.0)	0.001
**RPR and TPPA titers**		
Log_2_(titers of RPR)*	1.7 (1.2–2.6)	0.002
Log_2_(titers of TPPA)^†^	1.6 (1.2–2.3)	0.004

**Abbreviations:** CI = confidence interval; OR = odds ratio; RPR = rapid plasma reagin; TPPA = *Treponema pallidum* particle agglutination.

* RPR titers of pregnant women with syphilis = 1–128.

^†^ TPPA titers of pregnant women with syphilis = 80–40,960.

## Discussion

A specific World Health Organization strategic goal for the elimination of congenital syphilis is the prevention of transmission of syphilis from mother to child (*1*). In this study, the rate of syphilis transmission from mother to infant (17.4%) was substantially higher than the rate of 5.2% found in a study conducted in Shenzhen, China in 2010 (*9*). In the current study, approximately one third (52 of 155) of mothers received a diagnosis of syphilis at delivery, resulting in 21 infected infants, whereas in the Shenzhen study, women with syphilis diagnosed at delivery were excluded from the study. If the maternal syphilis cases diagnosed at delivery also were excluded from this study, the transmission rate would decline to 5.8% (six of 103).

In this study, late diagnosis of maternal syphilis during pregnancy was a significant risk factor for congenital syphilis because late diagnosis might lead to late treatment or no treatment during pregnancy. Penicillin is highly effective for the treatment of maternal syphilis and prevention of mother-to-child transmission (*7*). Thus, early syphilis screening, diagnosis, and treatment are important to prevent congenital syphilis and its associated adverse pregnancy outcomes. In addition, this study found that every twofold increase in maternal RPR titer nearly doubled the risk for delivering an infant with congenital syphilis, consistent with findings from another report that also found a twofold increased risk for transmission with each doubling of nontreponemal titers (*10*). Among 24 women with RPR titers ≥1:16, syphilis was transmitted to 16 (66.7%) of their infants, and among two women with RPR titers ≥1:64, both infants were infected. High titers of nontreponemal antibodies suggest early syphilis, including primary, secondary, and early latent syphilis. Therefore, patient and provider education about these infectious stages of syphilis could be beneficial. In addition, although treponemal antibody titers indicate exposure to syphilis and are not considered useful in the diagnosis and management of the disease, in this study, every twofold increase in TPPA titer was associated with a 60% increase in the odds of congenital syphilis. Treponemal titers might be helpful in assessing the risk for congenital syphilis in infants born to infected mothers.

Although migrant status was not a significant risk factor in the multivariable analysis, the findings from the univariate analysis did indicate a fivefold increase in risk for mother-to-child transmission of syphilis among migrant women. Among women with syphilis diagnosed at >36 weeks’ gestational age, three fourths were migrant women, and approximately half of pregnant migrant women received a diagnosis of syphilis after 36 gestational weeks, highlighting opportunities to improve prenatal care received by the migrant population.

The findings in this study are subject to at least three limitations. First, the small number of congenital syphilis cases and controls might have limited the power of the study to detect differences in risk that have been identified in previous studies (e.g., stage of maternal syphilis and current syphilis status of the pregnant women’s spouses). Second, because the original study was not designed to investigate risk factors for mother-to-child transmission of syphilis, information regarding behavioral characteristics, status of other sexually transmitted infections, and reasons for the absence of syphilis testing or treatment of pregnant women and their spouses was not collected. Finally, the study was conducted among persons in one city in China and might not be representative of the rest of the population.

The findings of this analysis indicate that late diagnosis of active maternal syphilis is a principal risk factor for congenital syphilis. Strengthening community and provider education about diagnosis and timely treatment of syphilis in pregnancy, particularly syphilis with high titers of *T. pallidum* antibodies, as well as expanding access to prenatal care services for migrant women might help prevent congenital syphilis. Further investigation into reasons for late diagnosis of syphilis in pregnant women in China, particularly among migrant women, also might help in developing policy for preventing congenital syphilis in China.

**Summary**

**What is already known about this topic?**

In China, the incidence of congenital syphilis increased nearly 26-fold from 2000 to 2013.

**What is added by this report?**

In a cohort of mothers with recently diagnosed syphilis, migrant women and those who received a diagnosis at >36 gestational weeks were approximately five times and 25 times more likely, respectively, to deliver an infected baby than were nonmigrant women and those who received a diagnosis earlier in pregnancy. Every twofold increase of maternal nontreponemal or treponemal antibody titers doubled the odds of delivering an infected infant.

**What are the implications for public health practice?**

Early diagnosis and treatment and improving access to prenatal care for migrant women are critical to preventing congenital syphilis in China.

## Footnotes

† Infants of adequately treated mothers received a single intramuscular dose of benzathine penicillin G (50,000 units/kg). Infants of inadequately treated or untreated mothers received a 10-day course of intravenous aqueous crystalline penicillin G (50,000 units/kg/dose) every 12 hours during the first 7 days of life and every 8 hours for the remaining 3 days.
